# MRI-based risk factors for intensive care unit admissions in acute neck infections

**DOI:** 10.1016/j.ejro.2025.100648

**Published:** 2025-04-01

**Authors:** Jari-Pekka Vierula, Harri Merisaari, Jaakko Heikkinen, Tatu Happonen, Aapo Sirén, Jarno Velhonoja, Heikki Irjala, Tero Soukka, Kimmo Mattila, Mikko Nyman, Janne Nurminen, Jussi Hirvonen

**Affiliations:** a Department of Radiology, Turku University Hospital, Turku, Finland; b Turku Brain and Mind Center, University of Turku, Turku, Finland; c Department of Otorhinolaryngology-Head and Neck Surgery, University of Turku and Turku University Hospital, Turku, Finland; d Department of Oral and Maxillofacial Surgery, University of Turku, Turku, Finland; e Medical Imaging Center, Department of Radiology, Tampere University and Tampere University Hospital, Tampere, Finland

**Keywords:** acute neck infection, magnetic resonance imaging, retropharyngeal edema, abscess, predictive model

## Abstract

**Objectives:**

We assessed risk factors and developed a score to predict intensive care unit (ICU) admissions using MRI findings and clinical data in acute neck infections.

**Methods:**

This retrospective study included patients with MRI-confirmed acute neck infection. Abscess diameters were measured on post-gadolinium T1-weighted Dixon MRI, and specific edema patterns, retropharyngeal (RPE) and mediastinal edema, were assessed on fat-suppressed T2-weighted Dixon MRI. A multivariate logistic regression model identified ICU admission predictors, with risk scores derived from regression coefficients. Model performance was evaluated using the area under the curve (AUC) from receiver operating characteristic analysis. Machine learning models (random forest, XGBoost, support vector machine, neural networks) were tested.

**Results:**

The sample included 535 patients, of whom 373 (70 %) had an abscess, and 62 (12 %) required ICU treatment. Significant predictors for ICU admission were RPE, maximal abscess diameter (≥40 mm), and C-reactive protein (CRP) (≥172 mg/L). The risk score (0−7) (AUC=0.82, 95 % confidence interval [CI] 0.77–0.88) outperformed CRP (AUC=0.73, 95 % CI 0.66–0.80, p = 0.001), maximal abscess diameter (AUC=0.72, 95 % CI 0.64–0.80, p < 0.001), and RPE (AUC=0.71, 95 % CI 0.65–0.77, p < 0.001). The risk score at a cut-off > 3 yielded the following metrics: sensitivity 66 %, specificity 82 %, positive predictive value 33 %, negative predictive value 95 %, accuracy 80 %, and odds ratio 9.0. Discriminative performance was robust in internal (AUC=0.83) and hold-out (AUC=0.81) validations. ML models were not better than regression models.

**Conclusions:**

A risk model incorporating RPE, abscess size, and CRP showed moderate accuracy and high negative predictive value for ICU admissions, supporting MRI’s role in acute neck infections.

## Highlights

• Soft tissue contrast of MRI provides prognostic biomarkers for acute neck infections.
• Risk score calculated from retropharyngeal edema, abscess size, CRP predicts ICU admissions.
• More evidence for the practical value of MRI in acute neck infections.

## 1 Introduction

Acute neck infections are potentially life-threatening medical emergencies that can lead to severe complications, such as airway obstruction and mediastinitis. The most common types are pharyngotonsillar and odontogenic infections [1], [4]. Imaging is used to confirm the diagnosis, detect drainable abscesses, and identify other potential complications [1]. In severe acute neck infections, treatment at the intensive care unit (ICU) may be indicated.

Imaging is often performed using contrast-enhanced computed tomography (CECT). However, CECT has limitations. Soft tissue contrast, even with an iodine intravenous contrast agent, is moderate at best [2], and the imaging involves ionizing radiation. Advanced techniques like dual-energy CT (DECT) may enhance iodine visualization and abscess detection. However, evidence comparing DECT to MRI in neck infections remains limited. Magnetic resonance imaging (MRI) has been shown to be more accurate than CECT for detecting infections and abscesses [3], and it is also feasible in the emergency setting [4]. MRI has been shown to be useful as the primary imaging modality in suspected acute neck infections [5]. MRI offers superb soft tissue contrast, thereby providing better visualization of soft tissue edema and abscess dimensions [4], [6], [7], [8], [9], [10].

Previously, the existence of specific edema patterns, such as retropharyngeal edema (RPE) and mediastinal edema (ME), has been demonstrated in many patients with acute neck infections [6]. These reactive edema patterns, visible as high signal intensity on fat-suppressed T2-weighted images, do not represent drainable fluid collections but rather markers of a severe course of illness. RPE, visible in about half of these patients, predicted ICU admissions in a preliminary evaluation, and ME, visible in about one-quarter of patients, predicted a longer duration of hospital stay [6]. In addition to these edema patterns, larger abscesses on MRI were associated with ICU admissions [6]. Taken together, many MRI-based biomarkers predict the severity of an acute neck infection, but their exact contributions to risk prediction and the optimal combination of risk factors are still inadequately understood.

This study sought to assess and quantify the contributions of MRI-based imaging findings to the risk of ICU admissions in a large cohort of patients with acute neck infections. Furthermore, it aimed to develop and test the classification performance of a combined risk score-based model for predicting ICU admissions, with the hypothesis that the risk score would outperform individual predictors. We complemented traditional statistical modeling with machine learning (ML) models and performed internal and hold-out validation to assess the robustness of the findings.

## 2 Methods

### 2.1 Patient selection

This is a retrospective single-center cohort study comparing emergency MRI findings with clinical outcomes in patients with acute neck infections. We followed previously published details about patient selection, MRI and its interpretation, and extraction of medical and surgical information [4], [6]. Permission was obtained from the hospital district board, and patient consent was waived due to the retrospective study setting. The inclusion criteria were: 1) emergency MRI between April 1, 2013, and August 30, 2021, for suspected neck infection; 2) MRI evidence of infection, that is, high signal on fat-suppressed T2-weighted Dixon images suggesting edema or high signal on fat-suppressed contrast-enhanced T1-weighted Dixon images suggesting abnormal tissue enhancement; 3) a final clinical diagnosis of an acute neck infection made by an otorhinolaryngologist or an oral and maxillofacial surgeon; and 4) adequate diagnostic image quality as determined by the radiologist reading the study. The exclusion criterion was the lack of clinical and surgical data.

### 2.2 MRI Protocol and Interpretation

Imaging was carried out with the Philips Ingenia 3 T System using a dS HeadNeckSpine coil (Philips Medical Systems, Best, Netherlands). The scan protocol included T2-weighted Dixon, T1-weighted Dixon, diffusion-weighted imaging (DWI), and post-gadolinium T1-weighted Dixon sequences, detailed in Table 3. RPE was defined as an area of a hyperintense signal of at least 2 mm in anteroposterior thickness between the prevertebral muscles posteriorly and the superior pharyngeal constrictor muscle anteriorly in at least two consecutive axial fat-suppressed T2-weighted Dixon images [6]. ME was defined as an area of a hyperintense signal in the soft tissues in axial fat-suppressed T2-weighted Dixon images at or below the level of the thoracic inlet, using the superior border of the manubrium sterni or the first thoracic vertebra as superior borders [6]. A gadolinium-based intravenous contrast agent (Dotarem®; Guerbet, Villepinte, France) was administered.

### 2.3 Clinical data

The following clinical and laboratory information was recorded for this study: age (years), sex (male/female), C-reactive protein (CRP) (mg/L), white blood cell count (WBC) (×10⁹/L), and treatment in the ICU (yes/no). ICU admission was determined by the responsible physician and was used as the reference standard in this study, regardless of specific admission criteria. In the context of acute neck infections, factors such as airway compromise, deep infection extension (e.g., mediastinitis), sepsis, and the need for intensive monitoring or surgical intervention are commonly associated with ICU admission. The mean interval between MRI and ICU admission was 4.6 hours (SD 4.4).

### 2.4 Statistical analysis

We first used multivariate binary logistic regression to identify significant independent predictors for ICU admission, which is considered clinical evidence of a severe course of disease. Predictors tested were age, sex, ME, RPE, WBC, CRP, and maximal abscess diameter. We split statistically significant continuous predictors into binary categories using a threshold value to assess regression coefficients. The Youden index was used to determine the optimal threshold value from receiver operating characteristic (ROC) curves. Statistically significant predictors from the regression analysis were included in the risk score-based model. Risk score coefficients were derived from regression coefficients by multiplying by two and rounding to the nearest whole number [11], and the total patient-level risk score was calculated as the sum of all predictor risk score coefficients.

Model performance was evaluated by the area under the curve (AUC) from ROC curves and their 95 % confidence intervals (CI). Individual predictors were also compared to model performance in a similar manner. The AUCs of the model and individual predictors were compared using the DeLong method [12]. P-values less than 0.05 were considered statistically significant. We also tested model performance as a binary classifier for ICU admission using optimal cutoff points determined by the Youden index. For this, sensitivity, specificity, positive predictive value (PPV), negative predictive value (NPV), and accuracy were calculated. Statistical analysis was conducted using IBM SPSS Statistics for Mac (version 26, copyright IBM Corporation 2019) and R (version 4.4.0) with the pROC package (version 1.18.5).

Additional analyses were performed with R (version 4.2.3) to complement those using traditional statistics. First, we employed four ML models: random forest (ranger package 0.16.0), XGBoost (xgboost package 1.7.8.1), support vector machine (SVM, e1701 package 1.7–13), and neural networks (NN, neuralnet package 1.44.2). Internal validation was achieved with 3-fold cross-validation and hold-out validation through a 60/40 split to training and testing datasets, stratifying with ICU admission. Validations were also performed for univariate statistics to compare the results to those from traditional statistics.

## 3 Results

The final cohort consisted of 535 patients (Table 1). This sample had a sex distribution of 57 % male and 43 % female and a mean age of 41 years (standard deviation [SD] 21). Pediatric patients (under the age of 18) made up 12 % of the sample. Of all patients, 373 patients (70 %) had an abscess. Altogether, 62 patients (12 %) were admitted to ICU.

**Table 1:** Patient characteristics.

**Characteristic**	**Value**
Number of patients	535
Age (years, mean ± SD)	41 ± 21
Female (N, %)	229 (43 %)
Pediatric (N, %)	66 (12 %)
Symptoms duration (days, mean ± SD)	5 ± 5
CRP (mg/L)	122 ± 86
WBC (× 10^9^/L)	14 ± 5
RPE (N, %)	276 (52 %)
ME (N, %)	129 (24 %)
Abscess (N, %)	373 (70 %)
Maximal abscess diameter (mm, mean ± SD)	24 ± 24
Surgery (N, %)	364 (68 %)
ICU (N, %)	62 (12 %)
Length of hospital stay (days, mean ± SD)	4 ± 4

CRP, C-reactive protein; ICU, intensive care unit; ME, mediastinal edema; N, number; RPE, retropharyngeal edema; SD, standard deviation; WBC, white blood cell count

RPE, maximal abscess diameter, and CRP were identified using multivariate binary logistic regression as statistically significant independent predictors for ICU admissions (Table 2). The cut-off points calculated from ROC curves with the Youden method for large abscess diameter and high CRP were 39.5 mm and 171.5 mg/L, respectively. Based on the significant predictors and their regression coefficients, the following risk score formula was derived: 3 *RPE (present/absent) + 2 * (CRP>171.5 mg/L, yes/no) + 2 * (abscess diameter >39.5 mm, yes/no). Risk scores had a range from 0 to 7.

**Table 2:** Multivariate binary logistic regression analysis for ICU admission.

	**Estimate**	Std. Error	P-value
**Age**	−0.005	0.008	0.487
**Sex**	−0.498	0.350	0.155
**ME**	0.595	0.356	0.094
**RPE**	1.398	0.446	**0.002**
**WBC**	0.047	0.030	0.125
**High CRP**	1.123	0.343	**0.001**
**Large abscess**	1.124	0.357	**0.002**

CRP, C-reactive protein; ME, mediastinal edema; RPE, retropharyngeal edema; WBC, white blood cell count

**Table 3:** MRI protocol for acute neck infections.

Sequence	Parameters	Scan time
T2 Dixon axial	Slice thickness 4 mm, TE= 100 ms, TR= 3021 ms, flip angle 90°	3 min 46 s
DWI axial	Slice thickness 4 mm, TE= 87 ms, TR= 3981 ms, b-value 1000 s/mm, flip angle 90°	48 s
T2 Dixon coronal	Slice thickness 3.5 mm, TE= 80 ms, TR= 3210 ms, flip angle 90°	5 min 14 s
T1 TSE axial	Slice thickness 4 mm, TE= 10 ms, TR= 641 ms, flip angle 90°	4 min 24 s
T1 Dixon axial post-contrast	Slice thickness 4 mm, TE= 7 ms, TR= 634 ms, flip angle 90°	3 min 29 s
T1 Dixon coronal post-contrast	Slice thickness 3.5 mm, TE= 14 ms, TR= 560 ms, flip angle 90°	3 min 8 s
T1 Dixon sagittal post-contrast	Slice thickness 3 mm, TE= 14 ms, TR= 630 ms, flip angle 90°	3 min 6 s
Total scan time		23 min 55 s

**Fig. 1:**
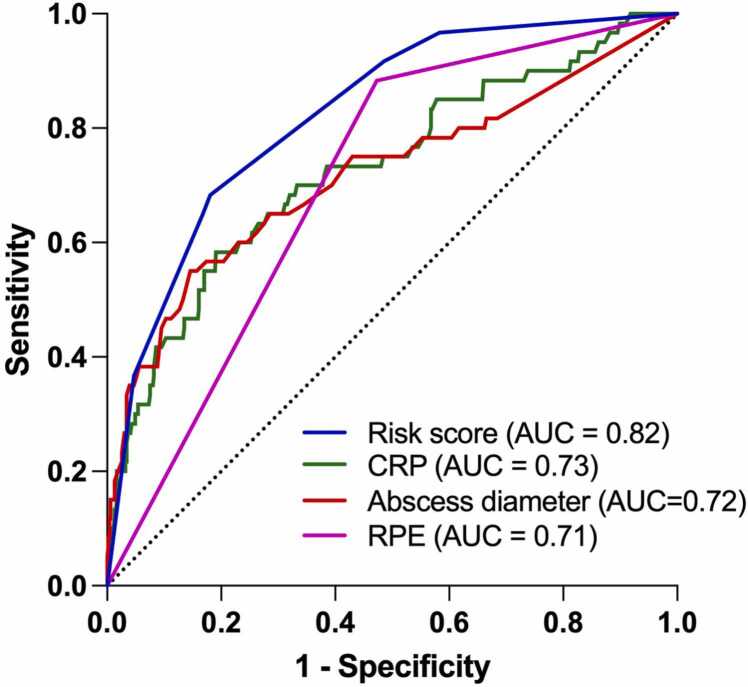
ROC curves for the risk score and the individual predictors for ICU admissions. Dotted line represents chance.

**Fig. 2:**
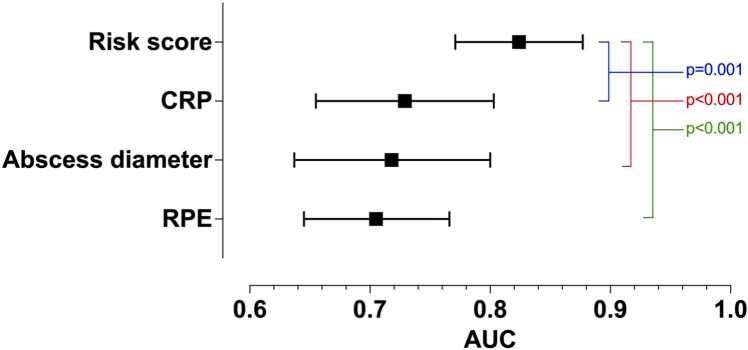
AUC values and their 95 % confidence intervals for the risk score and the individual predictors for ICU admissions.

**Fig. 3:**
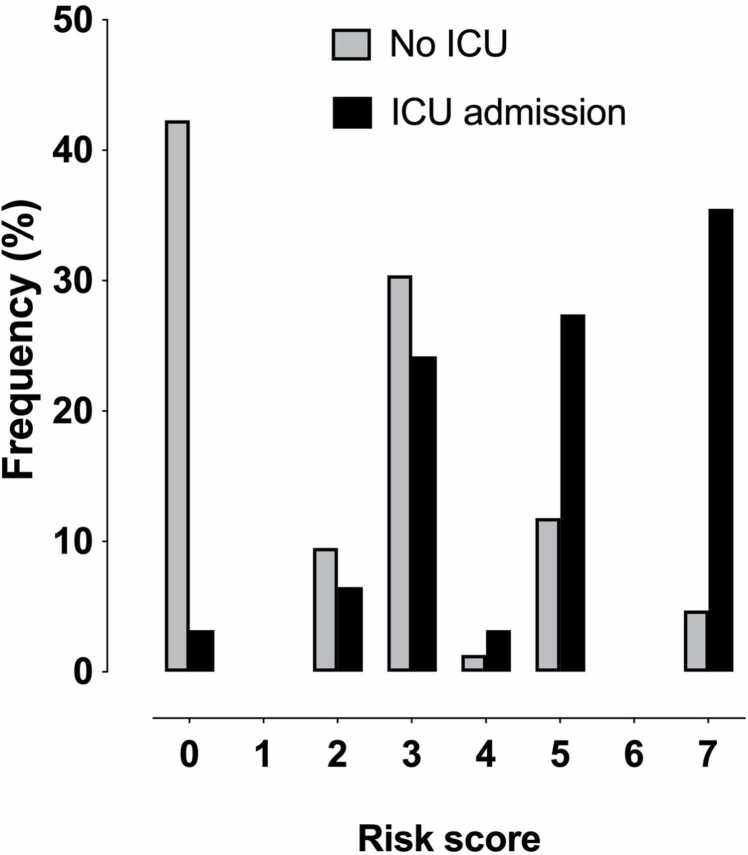
Frequency distributions of risk score values in patients admitted and not admitted to the ICU.

In the ROC analysis, the combined risk score model outperformed individual predictors (Fig. 1). The AUC value for the risk-score model was 0.82 (95 % CI 0.77–0.88). DeLong’s test showed that the risk-score model significantly outperformed the individual predictors: CRP (AUC=0.73, 95 % CI 0.66–0.80, *p* = 0.001), abscess diameter (AUC=0.72, 95 % CI 0.64–0.80, *p* < 0.001), and RPE (AUC=0.71, 95 % CI 0.65–0.77, *p* < 0.001) (p-values for AUC comparisons) (Fig. 2). The risk score distributions are given in Fig. 3.

The combined risk score had a cut-off of > 3, meaning that the prediction was optimal when comparing patients with 0–3 points (low risk) against those with 4–7 points (high risk). This cut-off yielded a sensitivity of 66 %, a specificity of 82 %, a PPV of 33 %, an NPV of 95 %, an accuracy of 80 %, and an odds ratio of 9.0 (p < 0.001) for ICU admissions. The proportion of patients admitted to the ICU was 5 % in the lower-risk group and 33 % in the higher-risk group. Assessing two major subtypes of neck infections, the odds ratio of the risk score was much higher in the 156 patients with odontogenic neck infections (40.0, *p* < 0.001) than in the 183 patients with pharyngotonsillar infections (5.0, *p* = 0.001). Regarding patient outcomes, high-risk patients had a longer average hospital stay (7 days, SD 6) than low-risk patients (3 days, SD 2) (p < 0.001).

In the internal validation, the AUCs of high CRP (0.70, 95 % CI 0.66–0.75), large abscess diameter (0.68, 95 % CI 0.44–0.91), RPE (0.73, 95 % CI 0.60–0.85), and risk score (0.83, 95 % CI 0.69–0.96) were comparable to those from the initial ROC analysis. The ML models did not yield significantly higher AUCs than the risk score model: random forest 0.81 (95 % CI 0.62–0.99), XGBoost 0.79 (95 % CI 0.61–0.97), SVM 0.66 (95 % CI 0.62–0.70), NN 0.80 (95 % CI 0.64–0.97). XGBoost and SVM were left out of hold-out validation due to inferior performance.

In the hold-out validation, ICU admission rates were similar in the training (11.1 %) and testing (10 %) datasets. In the testing dataset, comparable AUCs were obtained for high CRP (0.68), large abscess diameter (0.72), RPE (0.69), and risk score (0.81). Similarly, the AUCs from the ML models were stable in the testing dataset: random forest 0.81 and NN 0.80.

## 4 Discussion

In this large-scale cohort of MRI data from 535 patients with an acute neck infection, we show that high CRP (172 mg/L or higher), large abscesses (40 mm or larger), and RPE are significant risk factors for ICU admissions. Furthermore, a combined risk score model (4 points or higher) based on these predictors yielded a moderate overall accuracy (80 %) for ICU admissions, outperforming individual predictors. Low-risk scores had a high NPV (95 %). These effects were robust in the internal and hold-out validations. ML models did not improve discriminatory performance compared with traditional statistical models. The practical value of this finding is that patients with no more than one of these risk factors are very unlikely to require ICU treatment. Although the decision to admit the patient to the ICU is based on several clinical variables not captured by our model, such as airway difficulties and overall infection severity, imaging-based risk scoring might provide early helpful insight into patient management and inform about the possible need for closer follow-up.

RPE is a specific edema pattern that was shown to predict a severe course of acute neck infection in a previous smaller patient sample [6]. The retropharyngeal space is a small potential deep neck space between the visceral/buccopharyngeal fascia anteriorly and prevertebral fascia posteriorly [13]. RPE can be seen on MRI as high signal intensity on fat-suppressed T2-weighted sequences by radiologists with substantial interobserver agreement [6]. The performance of CECT in detecting RPE is generally limited due to its moderate soft-tissue contrast [2], unlike MRI’s high sensitivity for edema [4]. There are currently no studies examining whether RPE could be reliably detected using CECT. Additionally, maximal abscess diameter (40 mm or larger) predicted ICU admissions. MRI has excellent diagnostic accuracy (0.96) for abscesses when surgical proof is used as the reference standard [4], and maximal abscess diameter has good interobserver agreement [6]. The diagnostic accuracy of CECT is lower, owing to the difficulties of differentiating between phlegmon and drainable abscesses [2]. These findings highlight the unique benefits of MRI in acute neck infection afforded by superior soft tissue characterization and add to the growing evidence supporting the utility of MRI in clinical practice in these patients.

Our results are comparable with those from previous studies [14], in terms of the accuracy of predictive models for ICU admissions in infectious diseases. A similar approach has been used to predict ICU admissions in acute exacerbation in chronic obstructive pulmonary disease and to predict ICU admission in COVID-19 patients [15], [16], [17]. We were able to find only one published study with prediction models for ICU admissions in patients with deep neck abscesses after surgical drainage [18]. In that study, high AUC was obtained for the nomogram (0.91) as well as for a ML model (0.94), with high CRP being a significant predictor as in our study [18]. Contrary to this previous work, we chose not to include exhaustive medical history data, vital signs, or postoperative findings and instead focused on the data that is mostly likely to be available immediately after imaging (basic infection laboratory findings and MRI findings) to improve generalizability and early identification of at-risk patients. That is, we intentionally focused on simplicity and easy implementation of our model. Our imaging-based risk model should not be viewed as a comprehensive model for ICU admissions but rather as a potential tool for early detection of at-risk patients who might benefit from closer follow-up. Future work may improve our model by adding more variables and demonstrating clinical benefit.

While our study is the largest published cohort of MRI studies from clinically and surgically validated cases of acute neck infections (N = 535 patients), it is not without limitations. Admission to the ICU is a decision contributed by several clinical factors not included in the present study. The model relies on two imaging biomarkers, RPE and maximum abscess diameters. While maximum abscess diameter is usually easy to measure, detecting RPE might be more challenging for readers, especially less experienced radiologists and clinicians. RPE assessment relied on an expert consensus of two neuroradiologist readers, for whom a substantial interobserver agreement has been established [6], but further studies are needed to validate this biomarker in a larger sample of readers across different competence and specialty levels. Also, this model did not account for the type or location of infections and abscesses. Yet, performance was demonstrated for two main types of infections separately (odontogenic and pharyngotonsillar). MRI may not be available in most institutions and may not be suitable for all patients. Although findings were robust in internal and hold-out validations, we did not have the opportunity to perform external site validation of our risk score because we are not aware of other published cohorts of emergency MRI data from patients with acute neck infections. Finally, as discussed above, we tested only a limited number of predictors.

In conclusion, we propose a combined risk score based on CRP, RPE, and maximal abscess diameter, predicting ICU admissions on acute neck infection patients with 80 % accuracy and 95 % NPV.

## Ethics approval and consent to participate

IRB authorization or a waiver for patient consent was not sought because it is not required by the national legislature for retrospective studies of existing data.

## Funding statement

This study was financially supported by the Sigrid Jusélius Foundation (website: https://www.sigridjuselius.fi/en/). The funders had no role in study design, data collection and analysis, decision to publish, or preparation of the manuscript.

## CRediT authorship contribution statement

**Sirén Aapo:** Data curation. **Happonen Tatu:** Data curation. **Heikkinen Jaakko:** Data curation, Conceptualization. **Merisaari Harri:** Validation, Formal analysis. **Nurminen Janne:** Writing – review & editing, Data curation, Conceptualization. **Vierula Jari-Pekka:** Writing – review & editing, Writing – original draft, Investigation, Formal analysis, Conceptualization. **Nyman Mikko:** Writing – review & editing, Investigation, Conceptualization. **Mattila Kimmo:** Conceptualization. **Soukka Tero:** Conceptualization. **Irjala Heikki:** Conceptualization. **Velhonoja Jarno:** Conceptualization. **Hirvonen Jussi:** Writing – review & editing, Visualization, Supervision, Project administration, Investigation, Funding acquisition, Formal analysis, Data curation, Conceptualization.

## Declaration of Generative AI and AI-assisted technologies in the writing process

Statement: During the preparation of this work the authors used ChatGPT-4 in order to improve language and readability. After using this tool/service, the authors reviewed and edited the content as needed and take full responsibility for the content of the publication.

## Declaration of Competing Interest

The authors declare the following financial interests/personal relationships which may be considered as potential competing interests: Jussi Hirvonen reports financial support was provided by Sigrid Jusélius Foundation. If there are other authors, they declare that they have no known competing financial interests or personal relationships that could have appeared to influence the work reported in this paper.

## Data Availability

Patient data cannot be publicly shared because of the national legislature on patient data.
